# Assembly and comparative analysis of four complete mitochondrial genomes of *Pulsatilla* species

**DOI:** 10.1186/s12870-025-07527-1

**Published:** 2025-10-23

**Authors:** Yanping Xing, Che Bian, Jie Wu, Hefei Xue, Wenxiao Men, Wenjuan Hou, Yutong Huang, Yanchang Huang, Han Zheng, Jianhua Wang, Tingguo Kang, Yanyun Yang, Liang Xu

**Affiliations:** 1 School of pharmacy, Liaoning University of Traditional Chinese Medicine, Dalian, 116600 China; 2 School of Public Health, Shenyang Medical College, Shenyang, Liaoning 110034 China; 3 Liaoning Medical Functional Food Professional Technology Innovation Center, Shenyang, Liaoning 110034 China; 4 National Resource Center for Chinese Materia Medica, China Academy of Chinese Medical Sciences, Beijing, 100700 China; 5 State Key Laboratory of Dao-di Herbs, Beijing, 100700 China

**Keywords:** *Pulsatilla*, Evolutionary relationship, Mitogenome structure, Repeat sequence, Traditional chinese medicine

## Abstract

**Background:**

*Pulsatilla* species, which belong to the Ranunculaceae family, have garnered significant attention due to their remarkable medicinal attributes and ornamental value. In the present study, four mitochondrial genomes (mitogenomes) of *Pulsatilla* species were assembled and analyzed. The aim was to lay a research foundation for unraveling the genetic interrelationships among these species and the identification of Traditional Chinese Medicine from *Pulsatilla* species.

**Results:**

The mitogenomes of *P. chinensis*, *P. chinensis* var. *kissii*, *P. cernua*, and *P. dahurica* were assembled into single circular DNA molecules, with lengths of 878,988 bp, 684,203 bp, 747,621 bp, and 824,625 bp, encoding 53, 40, 40, and 49 protein-coding genes, 13, 14, 20, and 33 transfer RNA genes, and 3, 3, 4, and 3 ribosomal RNA genes, respectively. Repeat sequence analysis found a large number of simple sequence repeats (SSRs) and long repeat sequences (LRSs) in four mitogenomes. *P. chinensis*, *P. chinensis* var. *kissii*, *P. cernua* and *P. dahurica* had 43, 4, 6, 3 LRSs longer than 1 kb, respectively. Codon bias and RNA editing sites in the protein-coding genes of the four mitogenomes were similar. Furthermore, numerous collinear sequences were identified among the four mitogenomes, and homologous fragments were found by comparing them with their plastome sequences. Based on the phylogenetic trees of mitochondrial PCGs, *P. chinensis*, *P. chinensis* var. *kissii*, *P. cernua*, and *P. dahurica* clustered into a common subclade.

**Conclusions:**

The comprehensive analysis of the four *Pulsatilla* mitogenomes revealed that the genome size, gene composition, and distribution of repeat sequences display variability. This finding offers novel perspectives into the evolution of related species, thereby enriching our understanding of their genetic underpinnings and potential for further exploration in diverse fields.

**Supplementary Information:**

The online version contains supplementary material available at 10.1186/s12870-025-07527-1.

## Introduction

The genus *Pulsatilla* (Ranunculaceae) comprises approximately 38 species across Europe and Asia, with 11 species found in China [1, 2]. *Pulsatilla* plants called Pasque-flower have horticultural and medical importance [3, 4]. *Pulsatilla chinensis* (Bge.) Regel is a commonly used medicinal plant in China. Its roots, known as Pulsatilla radix in traditional Chinese medicine, have been included in the Chinese Pharmacopeia and have been utilized for over 2,000 years to clear away heat and toxins, cool blood, and treat dysentery [5]. Recent pharmacological research showed that its root extracts and polysaccharides exhibit therapeutic effects against cancer and leukemia [6–8]. However, roots of *P. chinensis* var. *kissii* (Mandl) S. H. Li & Y. H. Huang, *P. dahurica* (Fisch. ex DC.) Spreng. and *P. cernua* (Thunb.) Bercht. & J. Presl. are often misidentified as Pulsatilla radix due to morphological similarity and traditional use. Despite their medicinal properties, these species are considered substitutes. Research has demonstrated chemical differences among *P. chinensis*, *P. cernua*, and *P. dahurica* [9], and only *P. chinensis* and *P. chinensis* var. *kissii* contain anemoside B4 at levels meeting the standards of the Chinese Pharmacopoeia —variations that may impact therapeutic efficacy and safety [5, 10]. Accurate species identification is therefore essential for the proper use of these medicinal plants. *P. chinensis* var. *kissii* grows on dry slopes in Liaoning, China. Its basionym, *Pulsatilla kissii* Mandl (1922), has been suggested as a hybrid between *P. chinensis* and *P. cernua* [2], though no definitive evidence supports this claim, although there are reports of hybridization in *Pulsatilla* species [11]. Chloroplast gene analyses suggest a close relationship between *Pulsatilla* and *P. chinensis* var. *kissii* [12, 13], and molecular identification utilizing ITS2 has proven useful for species discrimination in some *Pulsatilla* species [14]. Other DNA barcodes *(rbcL*, *trnH*-*psbA*, *matK*, and ITS) have also been used in phylogenetic studies, though results have been somewhat inconsistent [15]. Expanding the genomic dataset may improve species discrimination and clarify phylogenetic relationships, thus aiding in the development and application of *Pulsatilla* medicinal resources.

The mitochondrion is an organelle derived from prokaryotic endosymbiont ancestors, and it plays an integral role in cell growth and cell development, impacting overall plant growth and development [16, 17]. For the most part, in seed plants, the mitochondrial genes are inherited maternally. The mitogenome demonstrates a high level of conservation and can function as a foundation for species classification, thereby resolving the issue of distinguishing between closely related species [18]. However, with the increasing reports on plant mitogenomes, numerous studies have revealed that shows notable variability in size, structure, and repeat content even among closely related species, offering valuable insights into plant evolution [19–22]. Meanwhile, mitochondrial genes are associated with plants’ environmental adaptability, tissue growth specificity, and fertility [15, 23–25]. They can also be used to identify the original animal and plant sources of Chinese medicinal materials, showing potential for the development of DNA molecular markers [24, 26, 27]. Therefore, research on mitogenomes can provide new genomic resources for studies on the phylogenetic relationships, evolution, and medicinal material identification of *Pulsatilla* plants.

This study presents four complete mitogenomes of the *Pulsatilla* species (*P. chinensis*, *P. chinensis* var. *kissii*, *P. cernua*, and *P. dahurica*) (Fig. S1) for the first time. The genome structure, gene composition, codon usage, RNA editing sites, and repeat sequences were investigated and analyzed. A phylogenetic analysis of 23 mitogenomes was undertaken based on 14 protein-coding gene (PCG) sequences. The goals were to clarify the characteristics of the mitogenomes of *P. chinensis*, *P. chinensis* var. *kissii*, *P. cernua*, and *P. dahurica*, as well as the differences among them. And by mitochondrial genes, the phylogenetic relationships and evolutionary positions of these species and variety will be explored. By analyzing the mitogenomes of these species, it can provide a research foundation for the environmental adaptation mechanisms of plants in the *Pulsatilla* genus and the improvement of cultivated varieties, while also offering basic data for developing molecular markers used in the identification of traditional Chinese medicines.

## Results

### General features of the sequenced mitogenomes of the four mitogenomes

**Fig. 1:**
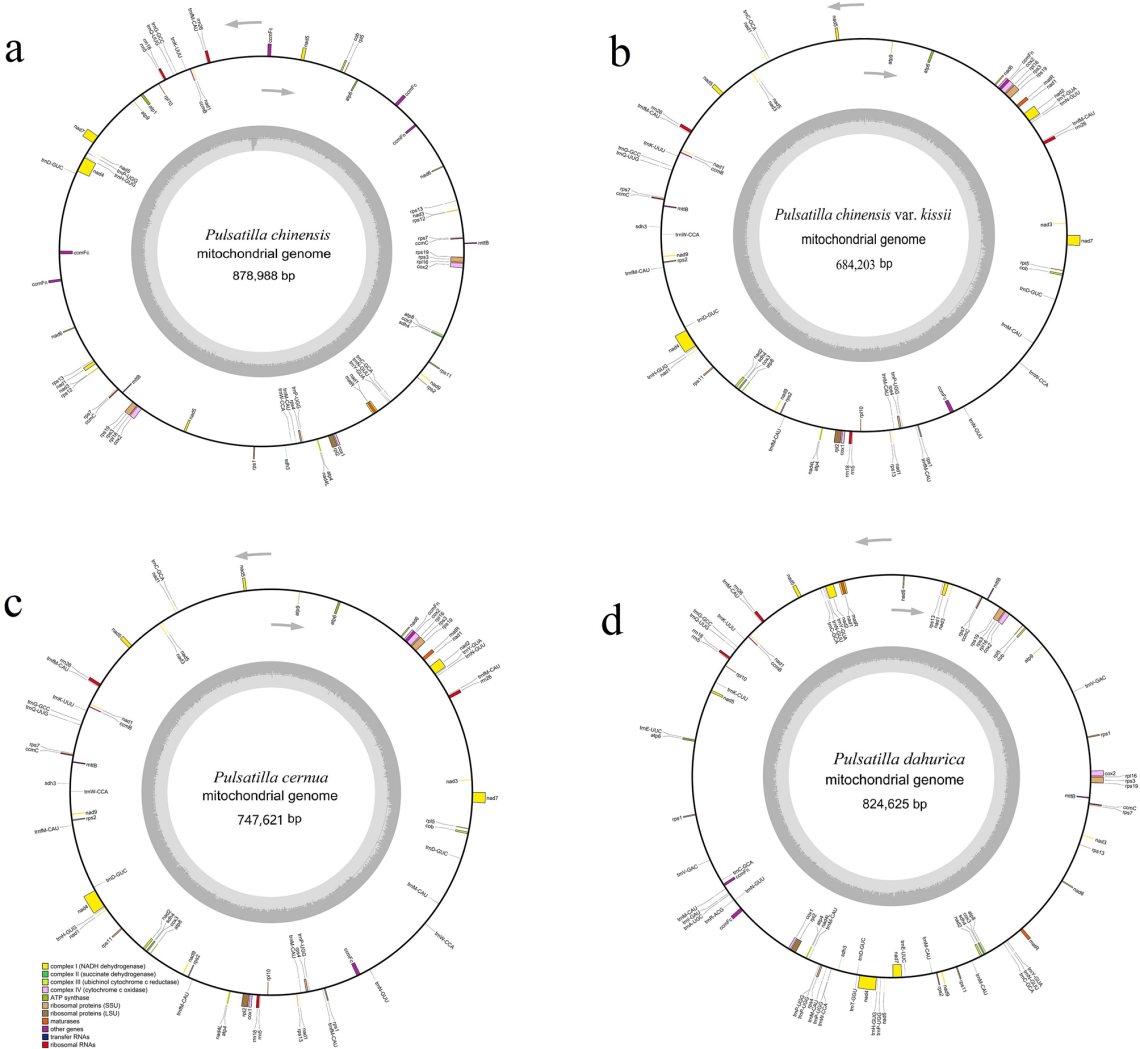
Circular maps of the *P. chinensis* (**a**), *P. chinensis* var. *kissii* (**b**), *P. cernua* (**c**), and *P. dahurica* (**d**) mitogenomes. Genes depicted on the outer and inner rings are transcribed in the clockwise and counterclockwise directions, respectively. Functional categories were indicated by color-coding

**Fig. 2:**
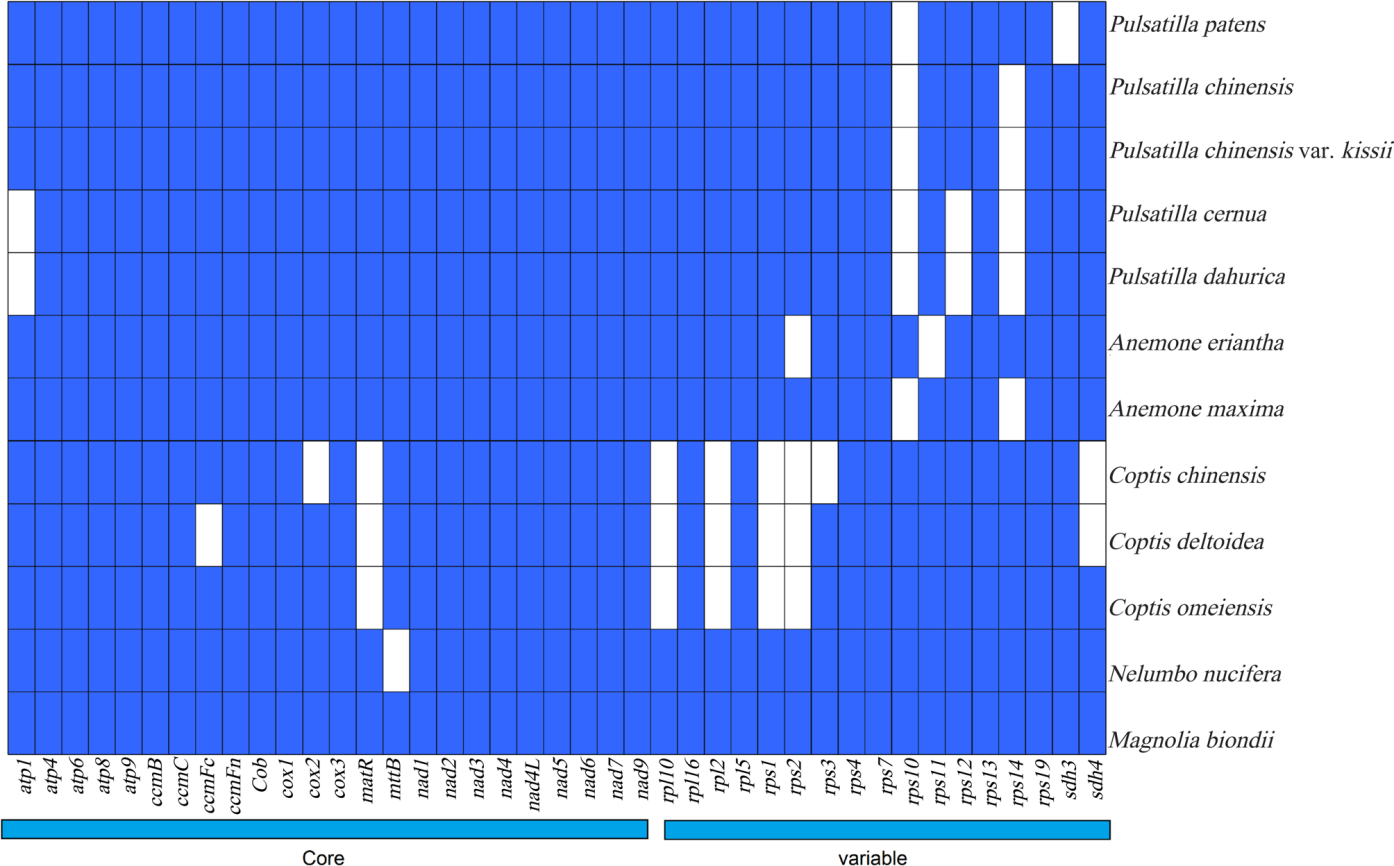
The presence of protein coding genes of mitogenomes in plants of the genus *Pulsatilla* and its related species, with blue boxes indicating the presence of those genes and white boxes indicating the absence of such genes. Core represented core genes and variable represented variable genes

The complete mitogenomes of *P. chinensis*, *P. chinensis* var. *kissii*, *P. cernua*, and *P. dahurica* were assembled as circular DNA molecules with lengths of 878,988 bp, 684,203 bp, 747,621 bp, and 824,625 bp, respectively and GC contents of 46.86%, 46.35%, 45.4%, and 46.46% (Fig. 1; Table 1). The average read coverage depth of the 4 samples ranged from 327.72X to 1243.97 (Fig. S2). All four mitogenomes were resolved to have a complete single circular molecular structure (Fig. S3). These genome sizes show considerable variation, differing notably from those of other *Ranunculaceae* species such as *Anemone maxima* and *Coptis* species [20, 28]. The number of PCGs identified in each mitogenome was 53 (*P. chinensis*), 40 (*P. chinensis* var. *kissii*), 40 (*P. cernua*), and 49 (*P. dahurica*) (Table S1). The genes a*tp1* and *rps12* were present in *P. chinensis* and *P. chinensis* var. *kissii* but absent in *P. dahurica* and *P. cernua*. According to the reported classification scheme [29], mitochondrial PCGs can be categorized into “core” and “variable” genes. The “variable” gene *rps10* was absent across all five *Pulsatilla* species (Fig. 2), highlighting the variability of gene content even among closely related taxa.

For all four mitogenomes, there were nine PCGs contained introns: *ccmFc*, *cox2*, *rpl2*, and *rps3* each harbored one intro; *nad1*, *nad2*, *nad5*, and *nad7* each contained four introns; and *nad4* contained three introns (Table S2). The number of tRNA genes was 13 in *P. chinensis*, 14 in *P. chinensis* var. *kissii*, 20 in *P. cernua*, and 33 in *P. dahurica*. Correspondingly, 3, 3, 4, and 3 rRNA genes were identified. Additionally, the number of open reading frames (ORFs) was 251 (*P. chinensis)*, 199 (*P. chinensis* var. *kissii*), 251 (*P. cernua*), and 241 (*P. dahurica*), with total ORF lengths of 119,223 bp, 92,253 bp, 138,696 bp, and 109,428 bp, respectively (Table S3).

**Table 1:** Summary of four mitogenomes sequencing statistics of *Pulsatilla* species

	*P*. *chinensis*	*P*. *chinensis* var. *kissii*	*P*. *cernua*	*P*. *dahurica*
Genome Size (bp)	878,988	684,203	747,621	824,625
GC Content (%)	46.86	46.35	45.4	46.46
protein-coding genes Number	53	40	40	49
Gene Total Length (bp)	47,436	35,433	35,085	42,996
tRNA (transfer RNA)	13	14	20	33
rRNA (ribosome RNA)	3	3	4	3
Gene Average Length (bp)	895	886	877	877
Gene’s GC Content (%)	43.96	43.83	43.65	44.17
% of Genome (Genes)	5.4	5.18	4.69	5.21
Intergenic region Length (bp)	831,552	648,770	712,536	781,629
Intergenic’s GC Content (%)	47.03	46.49	45.49	46.59
% of Genome (Intergenic)	94.6	94.82	95.31	94.79

### Repeat sequence analysis

**Fig. 3:**
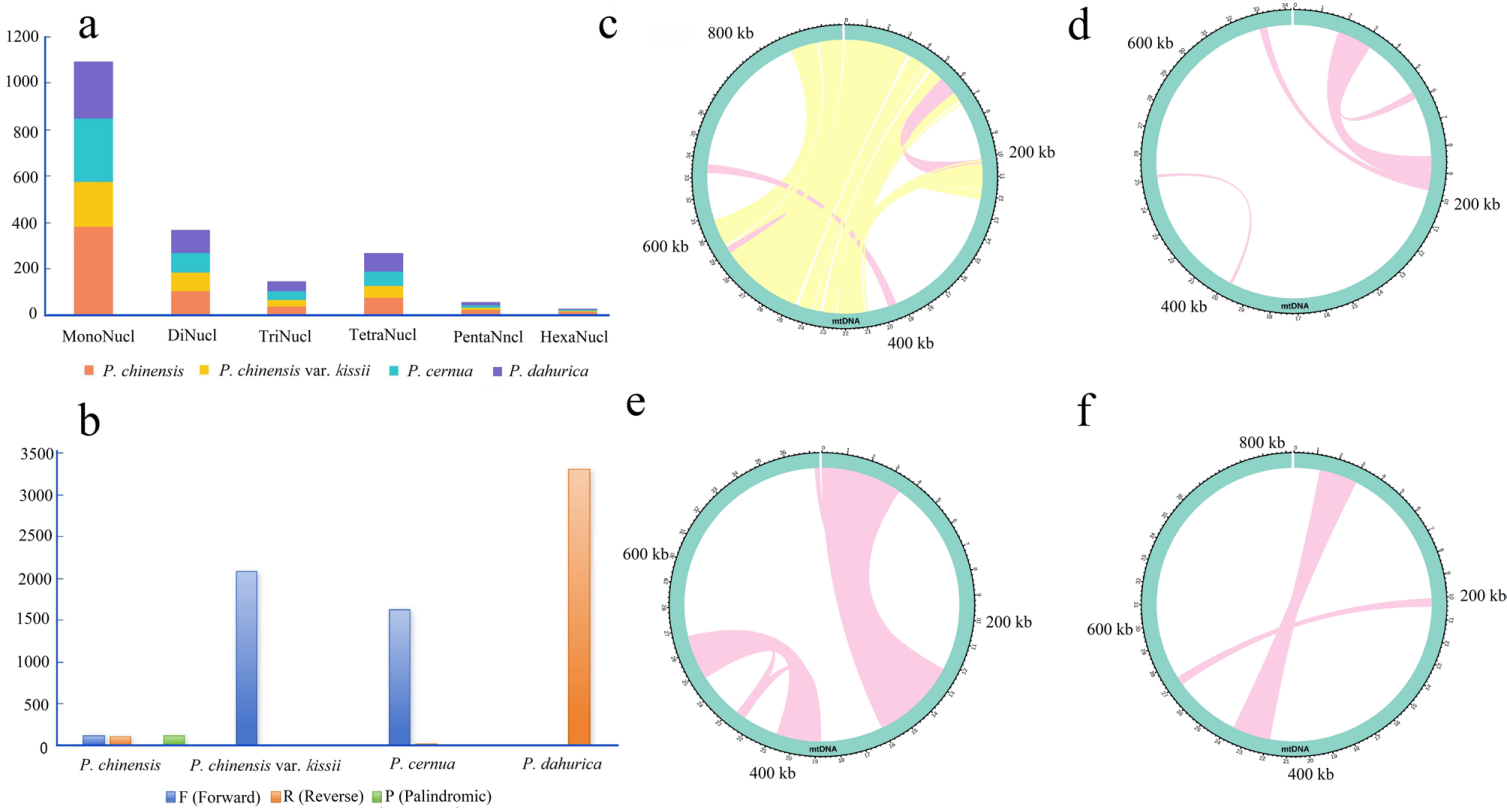
Repeats were detected in the mitogenomes of *P. chinensis*, *P. chinensis* var. *kissii*, *P. cernua*, and *P. dahurica*. **a**, Type and number of detected SSRs; **b**, Type and number of long repeat sequences. Repetitive sequences of more than 1 kb in the four mitogenomes: **c**, *P. chinensis*; **d**, *P. chinensis* var. *kissii*; **e**, *P. cernua*; **f**, *P. dahurica*; the yellow lines represented P (Palindromic), and the pink lines represented F (Forward) LRSs, respectively

Six types simple sequence repeats (SSRs) were identified in the mitogenomes of *Pulsatilla* species, with 631 in *P. chinensis*, 369 in *P. chinensis* var. *kissii*, 474 in *P. cernua*, and 481 in *P. dahurica* (Fig. 3a). Notably, *P. chinensis* uniquely harbored 41 SSRs exceeding 20 bp in length (Table S4). Across all four mitogenomes, monomeric SSRs were the most abundant, followed by dimeric SSRs and etrameric SSRs. The proportion of A/T in all SSRs was the highest, with 30.74%, 45.80%, 51.69%, and 44.28% in *P. chinensis*, *P. chinensis* var. *kissii*, *P. cernua*, and *P. dahurica*, respectively.

Long repeat sequences (LRSs) were also abundant in all four mitogenomes, categorized into forward (F), reverse (R), and palindromic (P) types (Fig. 3b). *P. dahurica* exhibited the highest number of LRSs (3,299 pairs), all of which were R-type. In contrast, *P. chinensis* (349 pairs) contained all three types. The length of LRSs ranges from 200 to 26,008 bp in *P. chinensis*, 30–21,799 bp in *P. chinensis* var. *kissii*, 30–71,126 bp in *P. cernua*, and 30–19,430 bp in *P. dahurica* (Table S5). *P. chinensis* contained 43 LRSs exceeding 1 kb, including eight over 10 kb and three over 20 kb. The other three species had significantly fewer LRSs over 1 kb. Notably, *P. cernua* had only six LRSs above 1 kb, yet included one over 70 kb and another over 30 kb (Fig. 3c-f).

### Codon bias ratios analysis

**Fig. 4:**
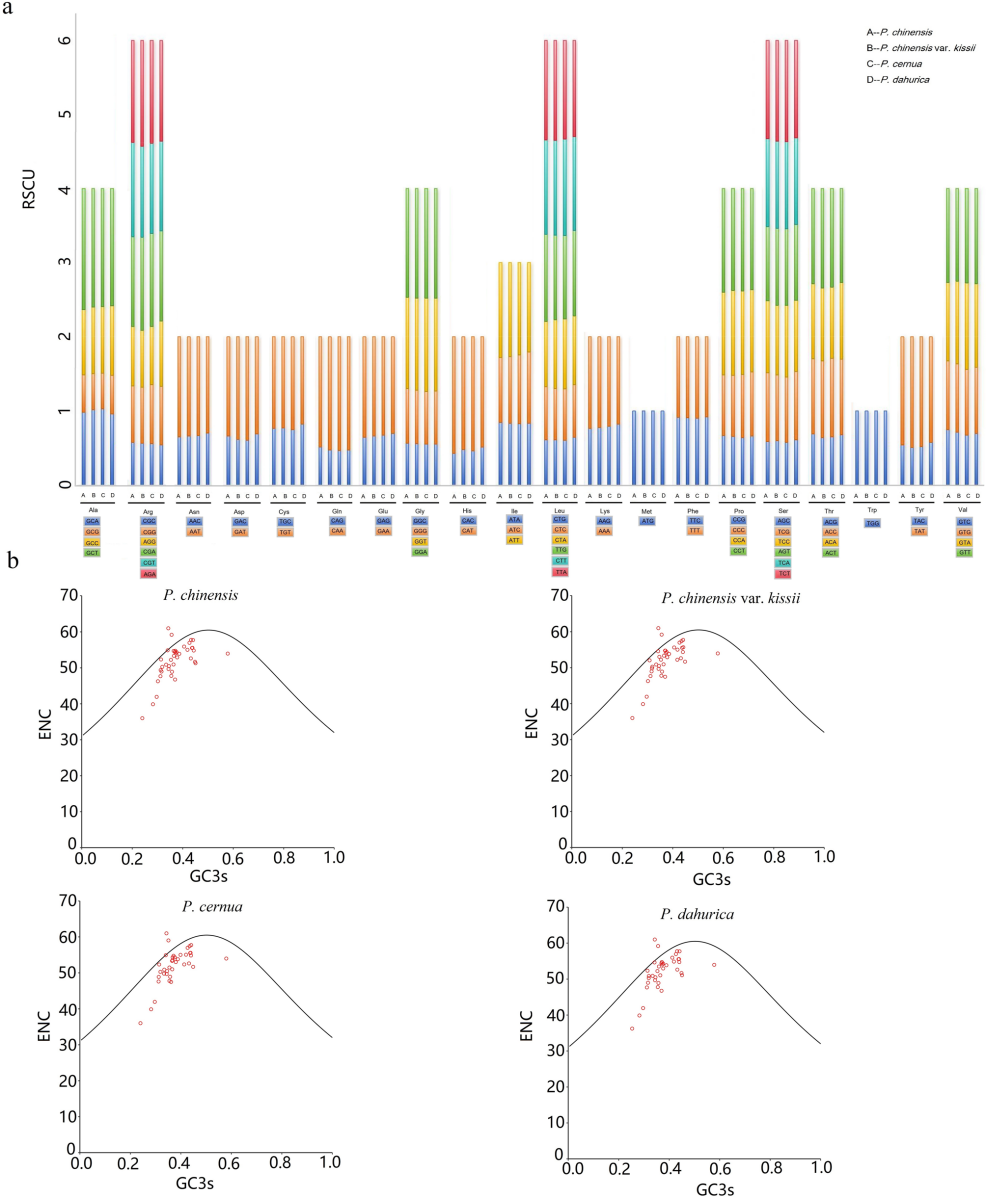
Codon bias and ENC plot of the PCGs in four mitogenomes of *Pulsatilla* species. **a**, RSCU values of the four mitogenomes; **b**, ENC plot of the four mitogenomes. Standard curve line was calculated as follows: ENC = 2 + GC3 + 29/(GC3s^2^ + (1-GC3)^2^)

The relative synonymous codon usage (RSCU) values were assessed for mitochondrial PCGs in the four *Pulsatilla* mitogenomes (Fig. 4a). Each mitogenomes contained exhibited 29 codons with RSCU values greater than 1, indicating codon usage bias. A strong preference was observed for A and T at the third codon position, while the first base showed a more balanced distribution. Among all codons, GCT (Ala) had the highest RSCU value (1.635). Overall, codon usage patterns were consistent across the four species, with minor exceptions: GCA (Ala) was more frequently used in *P. chinensis* var. *kissii* and *P. cernua*, while ACA (Thr) was favored in *P. chinensis* and *P. dahurica.* The start codon AUG (Met) and UGG (Trp) both maintained RSCU values of 1, aligning with patterns reported in other species [30, 31]. The effective number of codons (ENCs) revealed similar codon usage trends among the species (Fig. 4b and Table S6). Most PCGs clustered along the expected curve for 24–58% GC3. There were two PCGs, *nad9* and *sdh3*, whose ENC values were above the standard curve. A majority of the points lay below the standard curve, especially those of *rps13* and *atp9*, which were far below the standard curve and whose ENC values were less than 40.

###  RNA editing sites analysis

**Fig. 5:**
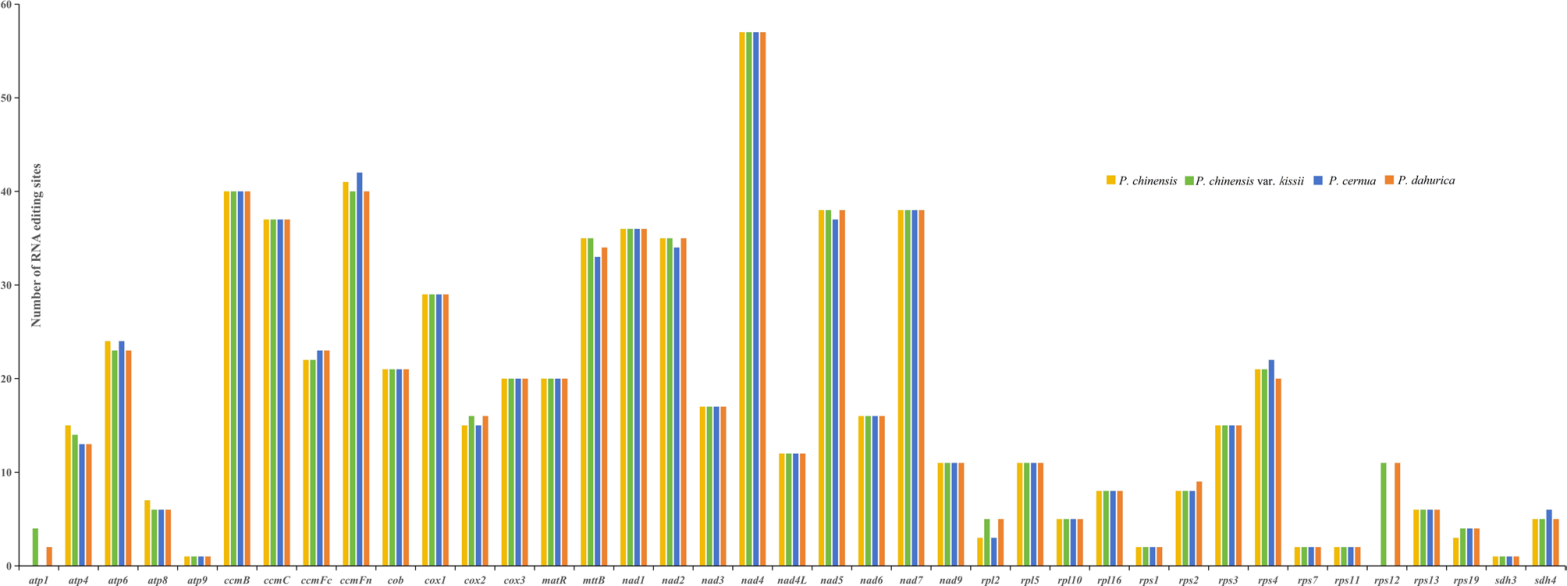
Distribution of RNA editing sites across the mitochondrial protein - coding genes of the four investigated plants

RNA editing refers to the addition, deletion, or replacement of nucleotides in RNA, causing changes in genetic information as the nucleotide sequences are different from corresponding genomic DNA sequences. This process occurs widely in organisms [32]. In the four *Pulsatilla* mitogenomes analyzed, RNA editing patterns in PCGs were largely consistent. A total of 691, 694, 679, and 677 RNA editing sites were identified in *P. chinensis*, *P. chinensis* var. *kissii*, *P. cernua*, and *P. dahurica*, respectively (Fig. 5). The number of editing sites varied by gene, from just one site in *atp9* to 57 in *nad4*. All editing events were cytosine-to-uracil (C-to-U) conversions. These edits led to the formation of start codons in *nad1*, *cox2*, *nad5*, and *atp4*, and stop codons in *atp6*, *rpl16*, and *ccmFC* (Table S7).

.

### Collinearity and plastid-derived region analysis

**Fig. 6:**
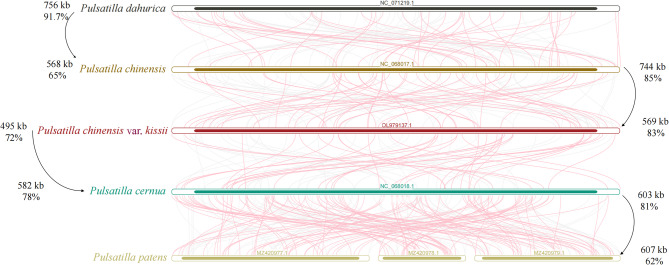
Multiple synteny plot of the five mitogenomes. Homologous sequences of more than 500 bp between two species were connected by arcs. Red arcs represented inverted sequences and gray arcs represented forward sequences

Sequence similarity analysis revealed extensive collinear blocks among the four *Pulsatilla* mitogenomes (Fig. 6). The collinear regions accounted for 65.0% (568 kb) and 91.7% (756 kb) of the total mitogenome length in *P. chinensis* and *P. dahurica*, respectively, with a total of 89 collinear blocks identified, including 24 blocks larger than 10 kb and 57 blocks between 1 and 10 kb in length. The collinear sequences accounted for 83.0% (569 kb) and 85.0% (744 kb) of the total sequences of *P. chinensis* var. *kissii* and *P. chinensis*, respectively, with a total of 100 collinear blocks identified, including 26 blocks larger than 10 kb and 67 blocks between 1 and 10 kb in length. Between *P. chinensis* var. *kissii* and *P. cernua*, there were 72 collinear blocks, with 23 longer than 10 kb and 46 longer than 1 kb. The collinear sequences accounted for 72.0% (495 kb) and 78.0% (582 kb) of the total sequences of *P. chinensis* var. *kissii* and *P. cernua*, respectively. The collinear regions accounted for 81.0% (603 kb) and 62.0% (607 kb) of the total mitogenome length in *P. patens* and *P. cernua*, respectively. Synteny analysis indicated a high degree of synteny conservation among the mitogenomes of species within the genus *Pulsatilla*. Gene-level collinearity showed more divergence than whole-genome comparisons (Fig. S4, Table S9). Between *P. chinensis* var. *kissii* and *P. chinensis*, 50 collinear blocks (PCGs) accounted for 82% and 62% of their total gene sequences, respectively. In contrast, the comparison between *P. chinensis* var. *kissii* and *P. cernua* revealed 36 collinear blocks (PCGs), comprising 74% and only 17% of the total gene sequences, respectively. The comparison between *P. chinensis* and *P. dahurica* identified just 18 collinear gene fragments (PCGs), each representing only 4% of their respective total gene sequence lengths.

**Fig. 7:**
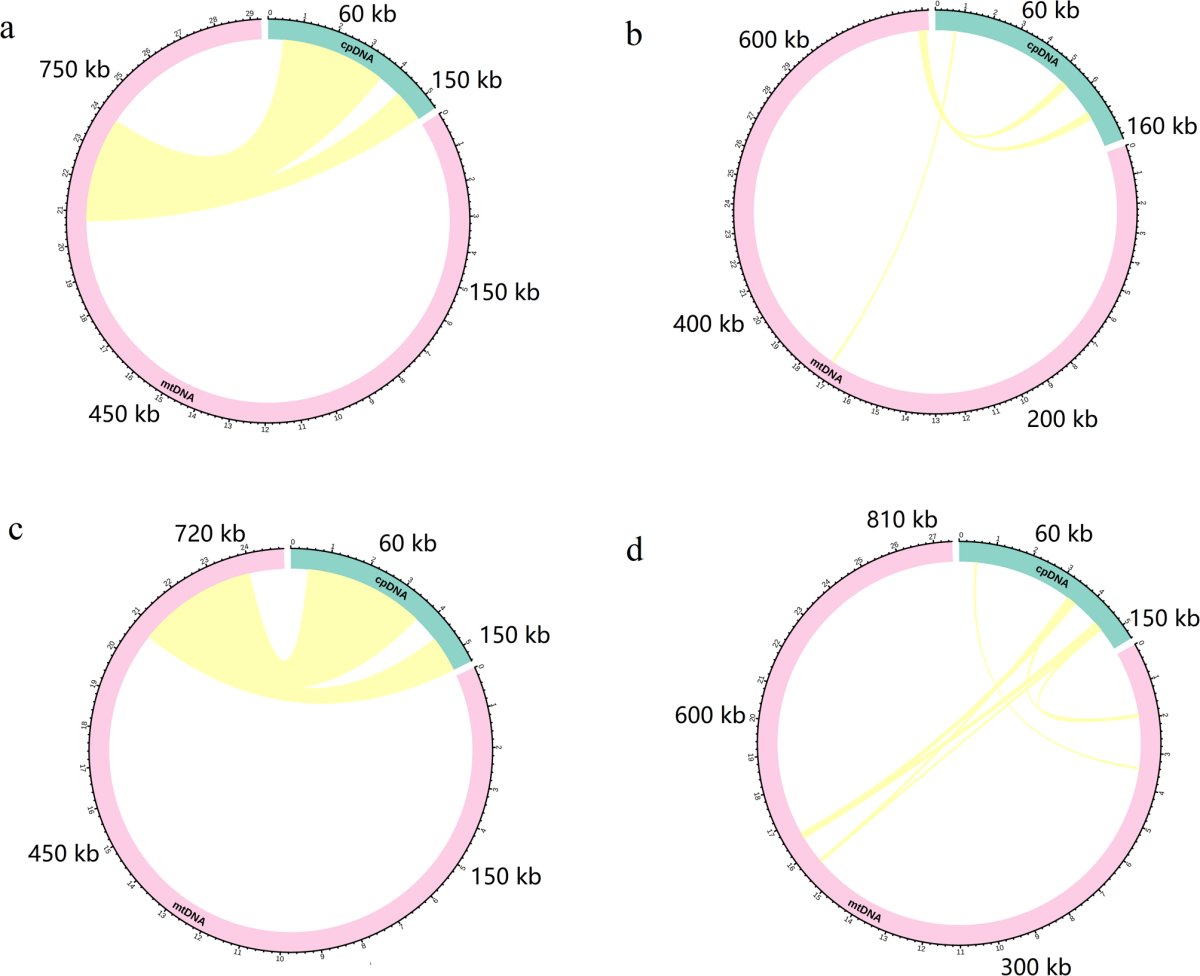
Plastid-derived region of mitochondrial sequences of the *Pulsatilla* species: **a**, *P. chinensis*; **b**, *P. chinensis* var. *kissii*; **c**, *P. cernua*; **d**, *P. dahurica*

A comparative analysis of mitochondrial plastid sequences (MTPTs) among the four *Pulsatilla s*pecies (Fig. 7) revealed that MTPT content and distribution varied notably: *P. cernua* contained 97 kb across 2 homologous fragments, *P. chinensis* had 19 kb (7 fragments), *P. dahurica* 13 kb (26 fragments), and *P. chinensis* var. *kissii* 7 kb (3 fragments). These MTPTs accounted for 13.1%, 2.0%, 1.6%, and 1.1% of the total mitogenome lengths, respectively.

### Phylogenetic analysis

**Fig. 8:**
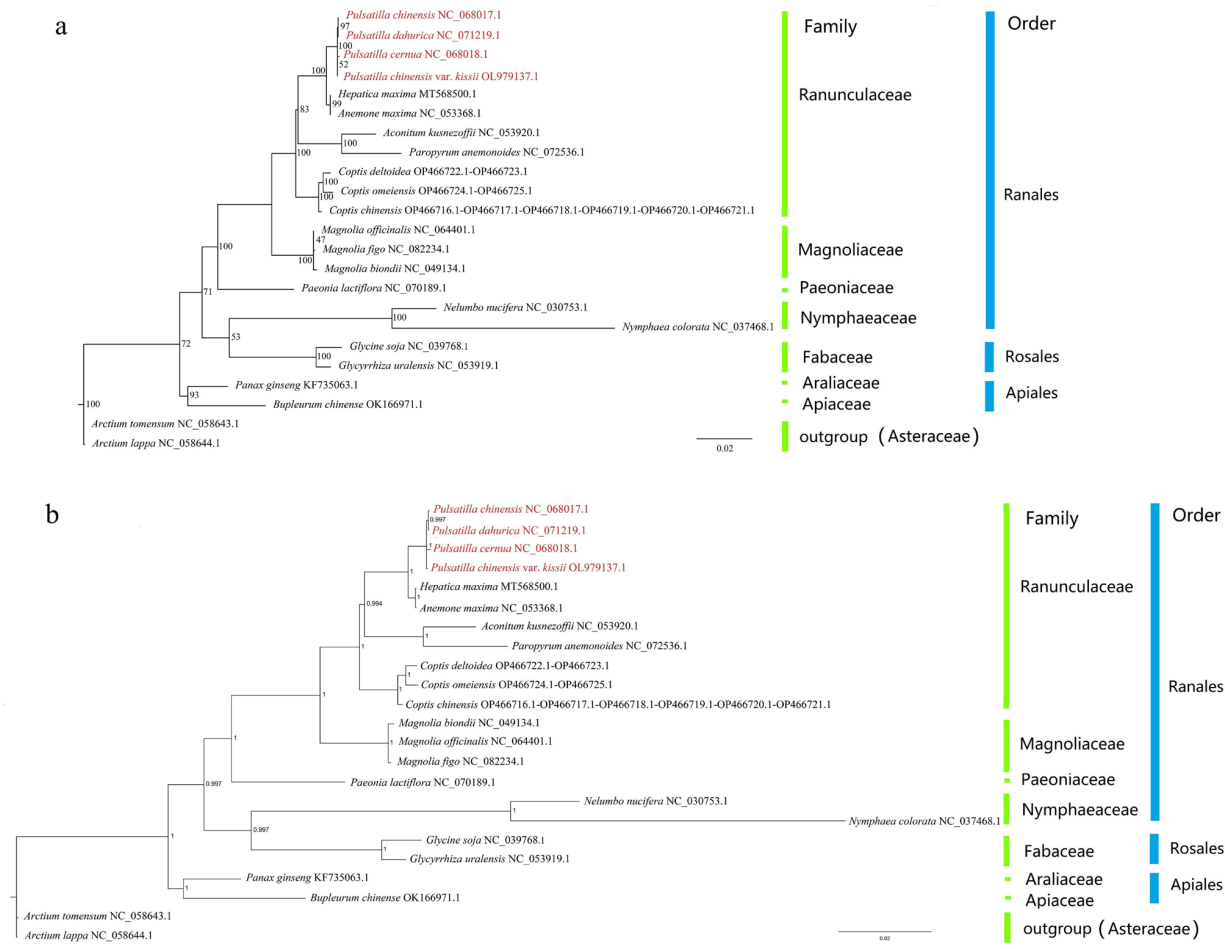
Phylogenetic trees constructed using 14 PCGs of 23 mitogenomes. **a**, Maximum likelihood (ML) tree; **b**, Bayesian inference (BI) tree

**Fig. 9:**
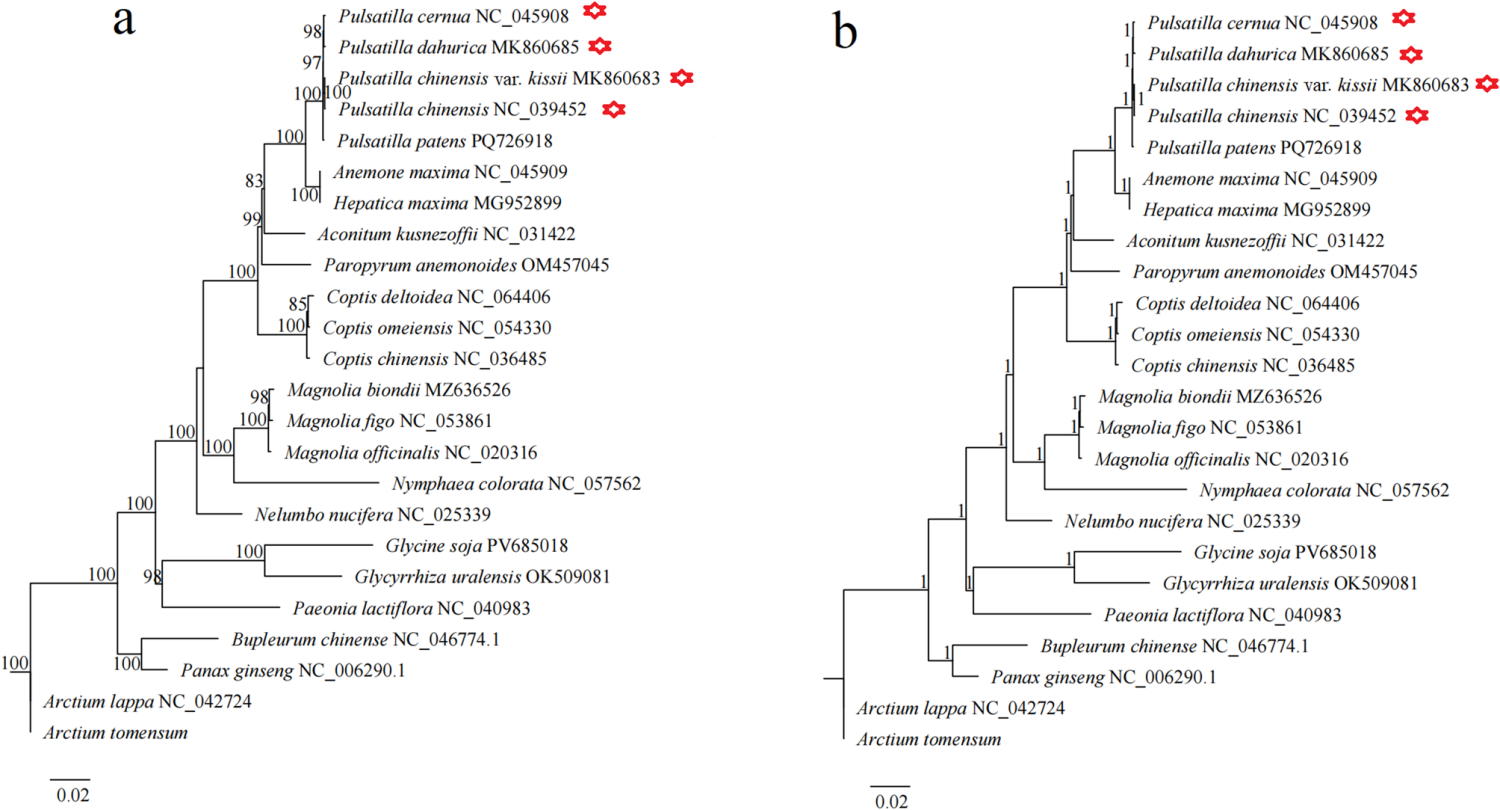
Phylogenetic trees constructed using 50 PCGs of 24 chloroplast genomes.** a**, Maximum likelihood (ML) tree; **b**, Bayesian inference (BI) tree

Phylogenetic trees constructed using maximum likelihood (ML) and Bayesian inference (BI) were largely congruent, with most families and orders forming monophyletic groups (Fig. 8). Within the Ranunculaceae family, *Pulsatilla* species formed a well-supported monophyletic clade. ML analysis grouped *P. chinensis* and *P. dahurica* together, while *P. chinensis* var. *kissii* clustered with *P. cernua*, though with weak support (52%) (Fig. 8a). In contrast, BI analysis grouped *P. chinensis* and *P. cernua*, with *P. chinensis* var. *kissii* and *P. dahurica* forming separate branches (Fig. 8b). Overall, *Pulsatilla* showed closer genetic affinity to *Anemone* and *Hepatica*. Gene concordance factor (gCF) and site concordance factor (sCF) analyses of the phylogenetic tree showed that this topological structure was not well-supported (Fig. S5). Our analysis of the shared protein sequences from four *Pulsatilla* species (used for constructing the phylogenetic tree in this study) revealed that these sequences were almost identical, which may be the reason why this phylogenetic tree cannot clarify the evolutionary relationships among species within *Pulsatilla*. Furthermore, we reconstructed ML and BI phylogenetic trees for *Hepatica maxima* and 4 *Pulsatilla* species using 21 conserved mitochondrial proteins. The results showed that the two phylogenetic trees exhibited different topological structures with low support values (Fig. S6). In contrast, the phylogenetic tree constructed using shared proteins from the chloroplast genome was consistent with previous research results (Fig. 9) [12, 13].

## Discussion

Plant mitogenomes are highly dynamic, with substantial variation in size, structure, and gene content—an ongoing focus of evolutionary research [33, 34]. In this study, the complete mitogenomes of *P. chinensis*, *P. chinensis* var. *kissii*, *P. cernua*, and *P. dahurica* were assembled. All four exhibited circular configurations, consistent with other species such as *Camellia duntsa*, *Hepatica maxima*,* Bupleurum chinense*, and *Avena longiglumis* [21, 28, 31, 35], but differing from *P. patens*, which has three linear mitochondrial chromosomes [1]. Such structural variation within genera has also been reported in *Rhodiola* and *Calla* [25, 36]. The mitogenome sizes of the four *Pulsatilla* species ranged from 684,203 bp to 878,988 bp—a nearly 100 kb difference—while that of *P. patens* extends to 986,613 bp [1], illustrating the size variation within the genus. The entire mitogenomes of four *Pulsatilla* species have a GC content ranging from 45.4% to 46.86%, which is relatively conserved compared with their genome sizes. Deletions of protein-coding genes (PCGs) are common in plant mitogenomes [29, 37], and *Pulsatilla* species contain between 40 and 53 PCGs, with gene number generally correlating with genome size [29]. The results demonstrated that compared with core genes, variable genes in the mitogenome are more prone to loss [38]. Consistent with earlier findings, the *rps10*, *rps14* gene is missing from all four *Pulsatilla* mitogenomes [1, 34] Notably, the core gene *atp1* and the variable gene *rps12* are absent in *P. dahurica* and *P. cernua*. These findings showed that the PCGs of mitogenomes among *Pulsatilla* exhibited diversity, potentially serving as the scientific references for species identification.

Plant mitogenomes are rich in repetitive sequences, which may contribute to their structural diversity and complexity [16, 39]. The *Pulsatilla* species analyzed in this study contained numerous repeats, including SSRs and LRSs. Each species harbored over 300 SSRs, with *P. chinensis* exhibiting the highest number, exceeding 600. Furthermore, in addition to significant differences in quantity (ranging from 369 to 631), the types of SSRs also showed variations (Table S4), and this phenomenon has also been reported in other species [38]. Multiple LRSs longer than 10 kb were identified, with significant variation in the number of LRSs >1 kb among species. *P. chinensis* had the highest LRS abundance, while *P. cernua* contained the longest LRS at 70 kb. LRSs are widespread within *Pulsatilla*, which may indicate extensive insertion and recombination events during the evolution of mitogenomes [24]. Such variability in repetitive sequence patterns may underlie the observed differences in mitogenome size and PCG content across *Pulsatilla* species [36].

The RSCU values reflects relationship between observed and expected codon frequencies, allowing assessment of species-specific codon preferences and evolutionary patterns [40]. In all four *Pulsatilla* mitogenomes, 29 codons showed RSCU values >1, most of which ended in A or T bases. This consistent codon preference aligns with patterns observed in other plant genera [35] and varies minimally among the *Pulsatilla* species. Similarly, ENC values were comparable across species [41, 42], indicating conserved codon usage bias within the genus. As in previous studies, most points fell below the expected curve in ENC-GC3s plots, suggesting that codon bias is primarily driven by natural selection or other non-neutral factors [43]. Notably, *Atp9* and *rps13* deviated substantially below the curve, reflecting stronger codon bias and higher mutational susceptibility relative to other genes [44].

RNA editing, a post-transcriptional modification present in all higher plants [32], helps maintain amino acid sequence conservation in essential mitochondrial proteins [45]. Our analysis showed that *P. chinensis* and *P. chinensis* var. *kissii* had more RNA editing sites in PCGs than *P. cernua* and *P. dahurica*. Each of the four mitogenomes contained over 600 editing sites (677–694), fewer than the 900 sites found in *P. patens* [1]. The distribution and pattern of RNA editing vary markedly among different losely related lineages [38]. C-to-U (C to T) editing dominated, consistent with other plant mitogenomes [1, 36]. RNA editing occurred in nearly all PCGs of *Pulsatilla*, likely playing a significant role in mitochondrial function [46]. These sites offer valuable targets for studying metabolic regulation of active compounds and stress responses in *Pulsatilla*.

Collinearity analysis, which examines homologous genes and sequence alignments, facilitates the study of evolutionary relationships among species [30]. The mitogenomes of the five *Pulsatilla* species exhibited extensive collinear blocks, reflecting high homology. Their evolution involved frequent sequence breaks and fusions [36]. Collinear regions accounted for 65% and 91.7% of the total mitogenome lengths in *P. chinensis* and *P. dahurica*, respectively, with the higher proportion in *P. dahurica* likely due to its abundance of LRSs, indicating that species with closer genetic relationships always share the majority of sequences [47]. Relatively, the existence of regions without homology highlights their uniqueness in this specific mitogenome, which holds important implications for subsequent studies related to genetics, growth, and development.

Gene transfer from plastids to mitochondria is a common evolutionary process in angiosperms [19, 47]. Among the four *Pulsatilla* mitogenomes, *P. cernua* contained 97 kb of MTPTs, representing 13.1% of its total mitogenome length—substantially higher than in the other three species. Although this exceeds the typical 1–10% range of plastid-derived regions in mitogenomes [48], similar proportions have been reported in three *Melastoma* species, with *M. dodecandrum* reaching approximately 15.16% [49]. This suggests that gene transfer from plastids to mitochondria may have occurred after *P. cernua* diverged from the other three species. The MTPTs may also contribute to the variation in *Pulsatilla* mitogenome size [48]. Additionally, mitogenome sequences can transfer back to plastomes [50, 51], indicating a dynamic, bidirectional gene exchange between mitochondria and chloroplasts rather than strict conservatism, even among closely related species.

Consistent with prior studies, species within the same genus or family clustered together in phylogenetic trees constructed from shared mitochondrial PCGs, reflecting congruence between traditional taxonomy and molecular data [22, 30]. The three *Pulsatilla* species and one variety formed a distinct clade. These results indicated that the mitogenome data can be effectively applied to distinguishing *Pulsatilla* species from species of other genera. Currently, mitogenome data, chloroplast genome data, and nuclear genome data are widely used in studies on species evolutionary relationships, but the results of these studies are often incongruent [4, 15]. Such phylogenetic incongruence among is well documented in evolutionary studies [38, 52–56] and may arise from processes including organelle capture (introgression), organelle genome recombination, and incomplete lineage sorting of ancient polymorphic organelle genomes [54]. Additionally, hybridization-driven independent organelle replacement can cause mitochondria and chloroplasts to carry differing parental evolutionary signals, leading to discordant phylogenies [15, 54]. For example, cucumber hybrid offspring inherit chloroplast genomes maternally but mitogenomes paternally [57]. However, our research results revealed that the PCGs in the mitogenomes of *Pulsatilla* plants provide low support for the evolutionary relationships among these four plant species. This may be because their protein sequences exhibit high conservation, such that insufficient variations have accumulated for species delimitation. This high conservation may partly result from the fact that the mutation rate of mitogenomes is generally lower than that of chloroplast genomes in plants [58]. If *P. chinensis* var. *kissii* was a hybrid, it also provides a plausible explanation for the species differentiation phenomenon in this genus [57]. In terms of mitogenome size and gene content, *P. chinensis* and *P. dahurica* share similarities, while *P. chinensis* var. *kissii* clusters with *P. cernua*. However, synteny analysis revealed that the mitogenome of *P. chinensis* var. *kissii* shows higher synteny with *P. chinensis* than with *P. cernua*. This discrepancy may be attributed to the relatively high level of MTPS in *P. cernua*. Although these findings do not conclusively prove a hybrid origin for *P. chinensis* var. *kissii*, mitogenomic data offer valuable insights into *Pulsatilla* phylogeny.

## Conclusions

We assembled the complete mitogenomes of *P. chinensis*, *P. chinensis* var. *kissii*, *P. cernua*, and *P. dahurica*, analyzing their genome organization, gene content, plastome-to-mitogenome horizontal gene transfer, and phylogenetic relationships. Considerable variation was observed in genome size (ranging from 684,203 bp in *P. chinensis* var. *kissii* to 878,988 bp in *P. chinensis*) and gene number (40 to 53 genes). Notably, *P. cernua* and *P. dahurica* lacked the *atp1* and *rps12* genes. All mitogenomes contained abundant repetitive sequences and homologous DNA fragments, with marked interspecific differences. Codon usage bias and RNA editing site patterns were similar across species. Phylogenetic analysis based on mitochondrial PCGs confirmed the monophyly of *Pulsatilla*. These results provide valuable genomic resources for plant mitogenome research and offer important insights into the genetic diversity and evolution of *Pulsatilla*, with potential applications in molecular breeding.

##  Materials and methods

### Sampling, DNA extraction, and sequencing

Fresh leaves of all four *Pulsatilla* species were collected from the Herb Garden of Dalian Campus, Liaoning University of Traditional Chinese Medicine, Dalian, Liaoning, China (39°06′N, 121°57′E) (Fig. S1). These plants, transplanted from their natural habitats, had been growing for over three years. According to the Regulations of the People^’^s Republic of China on Wild Plants Protection, these species are not classified as nationally key protected wild plants. Article five of the regulations stipulates that the scientific research on wild plants and in situ and ex situ protection of wild plants is encouraged and supported. All operations were carried out in accordance with guidelines in the Specification on Good Agriculture and Collection Practices for Medicinal Plants (GACP; Number: T/CCCMHPIE 2.1–2018). The study was approved by School of Pharmacy, Liaoning University of Traditional Chinese Medicine. The voucher specimens (*P. chinensis*, 20210503001LY; *P. chinensis* var. *kissii*, 20210503002LY; *P. cernua*, 20210503003LY; *P. dahurica*, 20210703011LY) were identified by Professor Liang Xu of Liaoning University of Traditional Chinese Medicine and deposited in the Chinese Medicine Specimen Hall of Liaoning University of Traditional Chinese Medicine. Mitochondria were isolated from leaves using density gradient centrifugation and treated with DNase I (Promega, Madison, USA) to remove genomic DNA contamination. Both short-read (Illumina) and long-read (PacBio Sequel II) sequencing technologies were employed. Approximately 2 µg of mitochondrial DNA was used to construct SMRTbell libraries with the PacBio Express Template Prep Kit 2.0. Libraries were multiplexed, size-selected with BluePippin (cutoff: 5000 bp), and sequenced on the PacBio Sequel II platform after annealing and binding via SMRT Link. For Illumina sequencing, approximately 1µg of mitochondrial DNA was sonicated to ~ 500 bp using the Covaris M220 system. The sonicated DNA was purified using a TIANgel Midi Purification Kit, and a sequencing library was constructed using the NEBNext^®^Ultra™ DNA Library Prep Kit for Illumina^®^ (New England Biolabs, Ipswich, MA, USA) according to the manufacturer’s instructions. Sequencing was performed on an Illumina NovaSeq 6000 with 150 bp paired-end reads. Short reads were quality checked using FastQC and trimmed with Trimmomatic (ILLUMINACLIP: TruSeq-PE.fa:2:30:10 LEADING:3 TRAILING:3 MINLEN:75). Long reads were base-called using Albacore v2.1.7 (mean_qscore > 7), demultiplexed by barcode, and converted to FASTA with Samtools Fasta (http://www.htslib.org/doc/samtools.html).

###  Genome assembly and annotation

The mitogenomes were assembled using two complementary strategies. The first involved de novo assembly of short clean reads with GetOrganelle v1.6.4, followed by extraction of mitochondrial contigs through BLAST v2.8.1 + alignment against mitochondrial protein-coding genes from the plant mitogenome database. Potential long mitochondrial reads were identified by mapping PacBio long reads to these contigs using BLASR v5.1 and subsequently assembled with Canu v2.1.1. The second strategy directly assembled all PacBio long reads using Canu. Draft contigs from both approaches were refined by mapping short clean reads using BWA and polished with Pilon v1.22. Circularity of contigs was confirmed using MUMmer 3.23, and the final corrected contigs from both methods were aligned with MUMmer for consistency. The assembly results were visualized and manually refined with Bandage v 0.8.1 software to obtain preliminary results [59]. Coverage depth was determined via Samtools (v 0.9) [60].

Mitochondrial gene annotation was conducted using the online GeSeq tool with default parameters to predict PCGs, transfer RNA (tRNA), and ribosomal RNA (rRNA) genes [61]. PCG locations were verified via BLAST against reference mitochondrial genes from *Liriodendron tulipifera* (GenBank accession MK340747.1). Start/stop codons and intron/exon boundaries were manually curated in SnapGene Viewer using the reference mitogenome. Mitogenome maps were generated with OGDRAW [62]. Functional annotation of genes involved BLAST searches (E-value ≤ 10⁻⁵) against several protein databases: NCBI Non-redundant (Nr), Swiss-Prot, Clusters of Orthologous Groups (COGs), Kyoto Encyclopedia of Genes and Genomes (KEGG), and Gene Ontology (GO). ORFs were identified using getorf (EMBOSS 6.6.0, http://emboss.sourceforge.net/) with parameters set to -table 1 and -size 300, corresponding to a minimum length of 100 amino acids.

### Sequence analysis

SSRs were identified using MISA software with parameters set for unit sizes and minimum repeats as follows: 1–8, 2–5, 3–4, 4–3, 5–3, and 6–3, and a minimum distance of 100 bp between SSRs. LRSs were detected using REPuter (https://bibiserv.cebitec.uni-bielefeld.de/reputer?id=reputer_manual_manual) with a minimum repeat length of 30 bp, Hamming distance of 3, and a maximum of 5000 repeats (equivalent to 1e-3). Four types of repeats were identified: forward (F), reverse (R), complementary (C), and palindromic (P). Codon usage bias was analyzed by calculating relative synonymous codon usage (RSCU) values using the cusp tool from EMBOSS v6.6.0. The effective number of codons (ENC) for protein-coding genes (PCGs) was evaluated via an online platform (http://cloud.genepioneer.com:9929/#/tool/alltool/detail/290). RNA editing sites were predicted using PREPACT3 (v3.12.0; http://www.prepact.de/) with a cutoff threshold of 0.2.

### Plastid-derived region analysis

Plastome sequences of the four species (*P. chinensis* MK860682, *P. chinensis* var. *kissii* MK860683, *P. cernua*MK860687, and *P. dahurica* MK860685) were retrieved from the NCBI database. Homologous sequences between each species’ mitogenome and plastome were identified using BLAST v2.10.1 with default parameters. The results were visualized using the Circos package via an online platform (http://210.22.121.250:35588/CloudPlatform/home).

### Collinearity analysis of *Pulsatilla* species

To investigate intergenomic rearrangements and evolutionary relationships, pairwise mitogenome comparisons of the five *Pulsatilla* species—including P. patens, whose mitogenome data were downloaded from NCBI (accession numbers: MZ420977, MZ420978, MZ420979)—were conducted using BLASTN (parameters: e-value 1e-5, word size 9, gap open 5, gap extend 2). Collinear blocks were defined as homologous sequences longer than 500 bp. Multiple synteny was visualized with MCScanX [63].

### Phylogenetic analysis

Mitogenomes of 17 closely related species and two outgroups (*Arctium lappa*, NC_058644; *A. tomentosum*, NC_058643) were selected from NCBI (https://www.ncbi.nlm.nih.gov) based on phylogenetic affinity. The mitogenome of *P. patens* was excluded due to unavailable annotation files. Using OrthoFinder v2.3.14, 14 conserved protein-coding genes (PCGs) were identified across the 23 mitogenomes: *atp9*, *nad6*, *nad3*, *cob*, *nad4*, *rpl16*, *ccmFn*, *atp8*, *nad7*, *atp4*, *nad2*, *cox3*, *ccmC*, and *ccmB*. Sequences were aligned with MUSCLE v3.8.1551 and refined using Gblocks to remove poorly aligned regions and gaps. The filtered alignments were concatenated for phylogenetic reconstruction. Bayesian inference was performed in MrBayes v3.2.6 using two hot and two cold chains for 10 million generations, sampling every 1,000 generations. The first 25% of trees were discarded as burn-in. Maximum likelihood (ML) analysis was conducted using IQ-TREEv1.6.12. The best-fit substitution model (HIVw + G + F) was determined by Modeltest v3.4 based on the Bayesian Information Criterion (BIC). Considering that ML and BI phylogenetic trees have similar topological structures in this study, we performed gene concordance factor (gCF) and site concordance factor (sCF) analyses on the ML phylogenetic tree, and calculated these factors using IQ-TREE v.2.4.0 [64]. Phylogenetic tree based on chloroplast genomes Fifty homologous single-copy protein-coding genes (including *accD*, *atpA*, *atpB*,* atpE*, *atpH*, *atpI*, *cemA*, *clpP*, *matK*, *ndhA*, *ndhC*, *ndhD*, *ndhE*, *ndhG*, *ndhI*, *ndhJ*, *petA*, *petB*, *petG*,* petL*, *petN*, *psaA*, *psaB*, *psaC*, *psaI*, *psbA*, *psbB*, *psbC*, *psbD*, *psbE*, *psbF*, *psbH*, *psbJ*, *psbK*, *psbT*, *psbZ*, *rbcL*, *rpl20*, *rpl33*, *rpl36*, *rpoA*, *rpoB*, *rpoC2*, *rps2*, *rps4*, *rps11*, *rps14*, *rps18*, *ycf3*) were selected from 24 samples via OrthoFinder v2.3.14. After individual alignment with MAFFT v7.429, ambiguous regions (gap-containing sites) were trimmed by Gblocks 0.91b, and sequences were concatenated for phylogenetic tree construction. Outgroups: *A. lappa* NC_058644 and *A. tomensum* (Download from the *Arctium lappa* database: http://210.22.121.250:41352/). ML tree: Constructed with IQ-TREE v1.6.1; best model (GTR + F + R3 via BIC) selected by ModelFinder, bootstrap = 1000. Bayesian tree: Built with MrBayes 3.2.6, using 2 hot + 2 cold chains, run for 10,000 generations, sampled every 1000 generations. First 25% trees discarded as burn-in, remaining used for consensus tree.

## Supplementary Information

Supplementary Material 1: Fig. S1 The four plants of *Pulsatilla* species Fig. S2 Sequencing depth and coverage map of the mitogenomes of *P. chinensis *(a), *P. chinensis *var*.kissii* (b), *P. cernua*(c), *P. dahurica *(d) Fig. S3 The assembly graph of mitogenomes displayed in Bandage. (a) Raw assembly networks showing complex reticulation; (b) Curated circular structures inferred from repeat masking and validation Fig. S4 Multiple synteny genes of the four mitogenomes Fig. S5 Maximum likelihood Phylogenetic trees constructed using 14 PCGs of 23 mitogenomes. The tree were annotated with supports which were indicated by concordance factors (UFB/gCF/sCF) Fig. S6 Phylogenetic trees constructed using 21 PCGs (*atp4*, *atp6*, *atp8*, *atp9*, *ccmB*, *cob*, *cox1*, *cox3*, *nad1*, *nad2*, *nad4*, *nad4L*, *nad5*, *nad7*, *rpl2*, *rpl5*, *rpl10*, *rps4*, *rps11*, *sdh3*, *sdh4*) of 5 mitogenomes. (a) Maximum likelihood (ML) tree; (b) Bayesian inference (BI) tree. 

Supplementary Material 2: Table S1 Genes predicted in the four mitogenomes of *Pulsatilla* species Table S2 The introns of protein-coding genes in four mitogenomes Table S3 The ORFs of four mitogenomes of* Pulsatilla* species Table S4 The SSR of four mitogenomes of *Pulsatilla* species Table S5 The Long repeat sequences of four mitogenomes of *Pulsatilla* species Table S6 ENC vaule of four mitogenomes of *Pulsatilla* species Table S7 The RNA-editing sites of four mitogenomes of *Pulsatilla* species. Table S8 Collinear blocks and their matched genes.

## Data Availability

The mitogenome sequences of P. chinensis, P. chinensis var. kissii, P. cernua, P. dahurica supporting the conclusions of this article are available in GenBank (https://www.ncbi.nlm.nih.gov/) with accession numbers NC_068017.1, OL979137.1, NC_068018.1, and NC_071219.1; BioProject accession numbers PRJNA786959, PRJNA787015, PRJNA787034, and PRJNA814807; Sequence Read Archive (SRA) data accession numbers SRR17163517 and SRR17163518, SRR17163616 and SRR17163617, SRR17173291 and SRR17173292, SRR18297520 and SRR18297521; BioSample accession numbers SAMN23765230, SAMN23765611, SAMN23765890, and SAMN26567201.
