# Supplementary material for: Assembly and comparative analysis of four complete mitochondrial genomes of Pulsatilla species
Source: BMC Plant Biol. 2025 Oct 23;25:1450. doi: 10.1186/s12870-025-07527-1 (PMC12548136; doi:10.1186/s12870-025-07527-1)
Supplement: Supplementary file 1 — Supplementary Material 1: Fig. S1 The four plants of Pulsatilla species Fig. S2 Sequencing depth and coverage map of the mitogenomes of P. chinensis (a), P. chinensis var.kissii (b), P. cernua(c), P. dahurica (d) Fig. S3 The assembly graph of mitogenomes displayed in Bandage. (a) Raw assembly networks showing complex reticulation; (b) Curated circular structures inferred from repeat masking and validation Fig. S4 Multiple synteny genes of the four mitogenomes Fig. S5 Maximum likelihood Phylogenetic trees constructed using 14 PCGs of 23 mitogenomes. The tree were annotated with supports which were indicated by concordance factors (UFB/gCF/sCF) Fig. S6 Phylogenetic trees constructed using 21 PCGs (atp4, atp6, atp8, atp9, ccmB, cob, cox1, cox3, nad1, nad2, nad4, nad4L, nad5, nad7, rpl2, rpl5, rpl10, rps4, rps11, sdh3, sdh4) of 5 mitogenomes. (a) Maximum likelihood (ML) tree; (b) Bayesian inference (BI) tree. [file 12870_2025_7527_MOESM1_ESM.docx]

Supplementary Figures

**
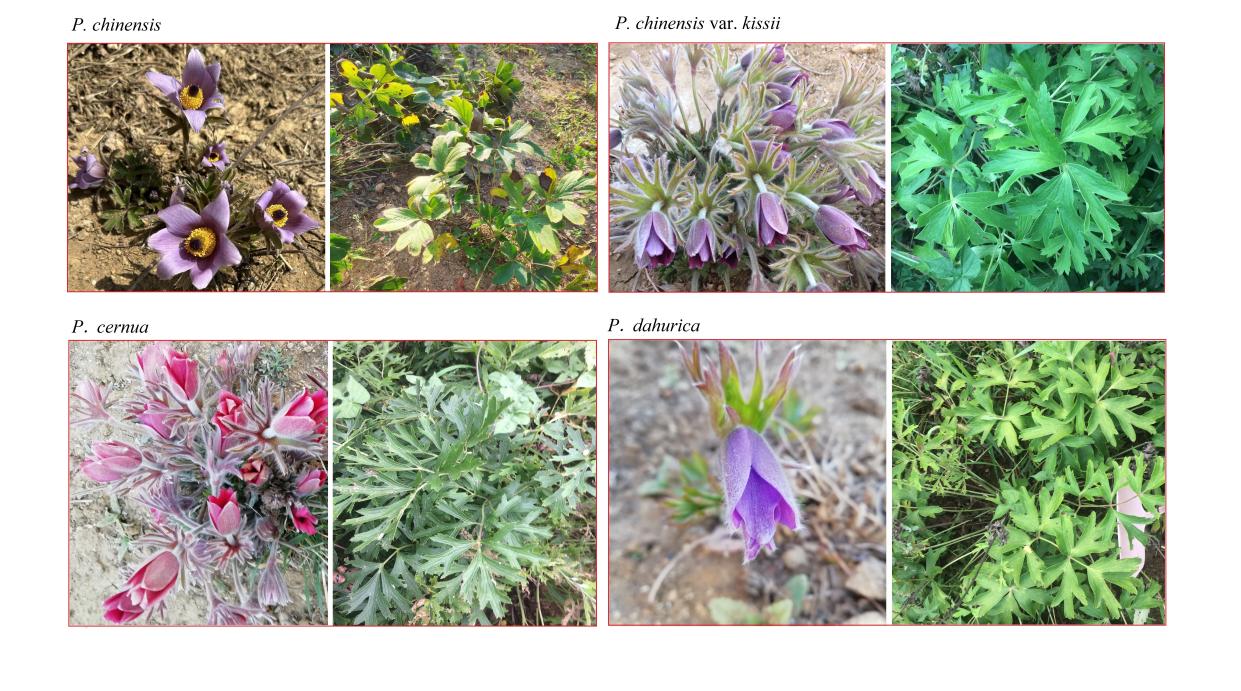
**

Fig. S1 The four plants of *Pulsatilla* species


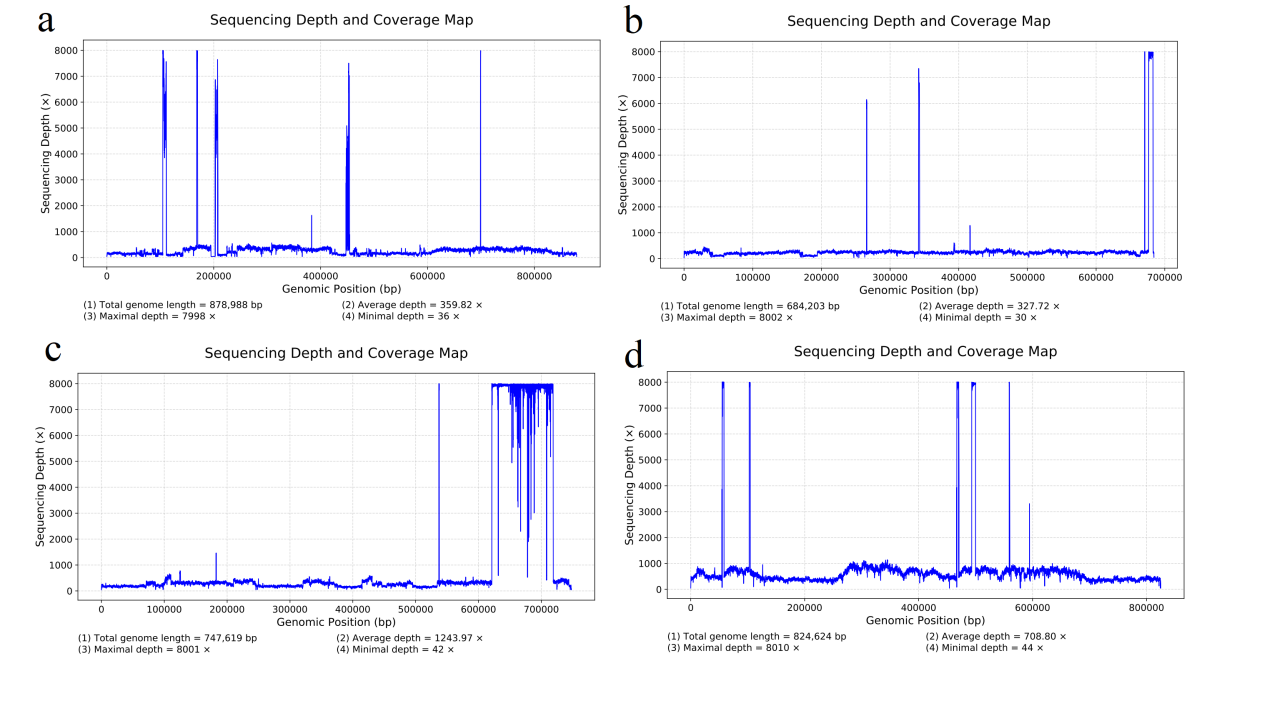


Fig. S2 Sequencing depth and coverage map of the mitogenomes of *P. chinensis* (a), *P. chinensis* var*. kissii* (b), *P. cernua* (c), *P. dahurica* (d)


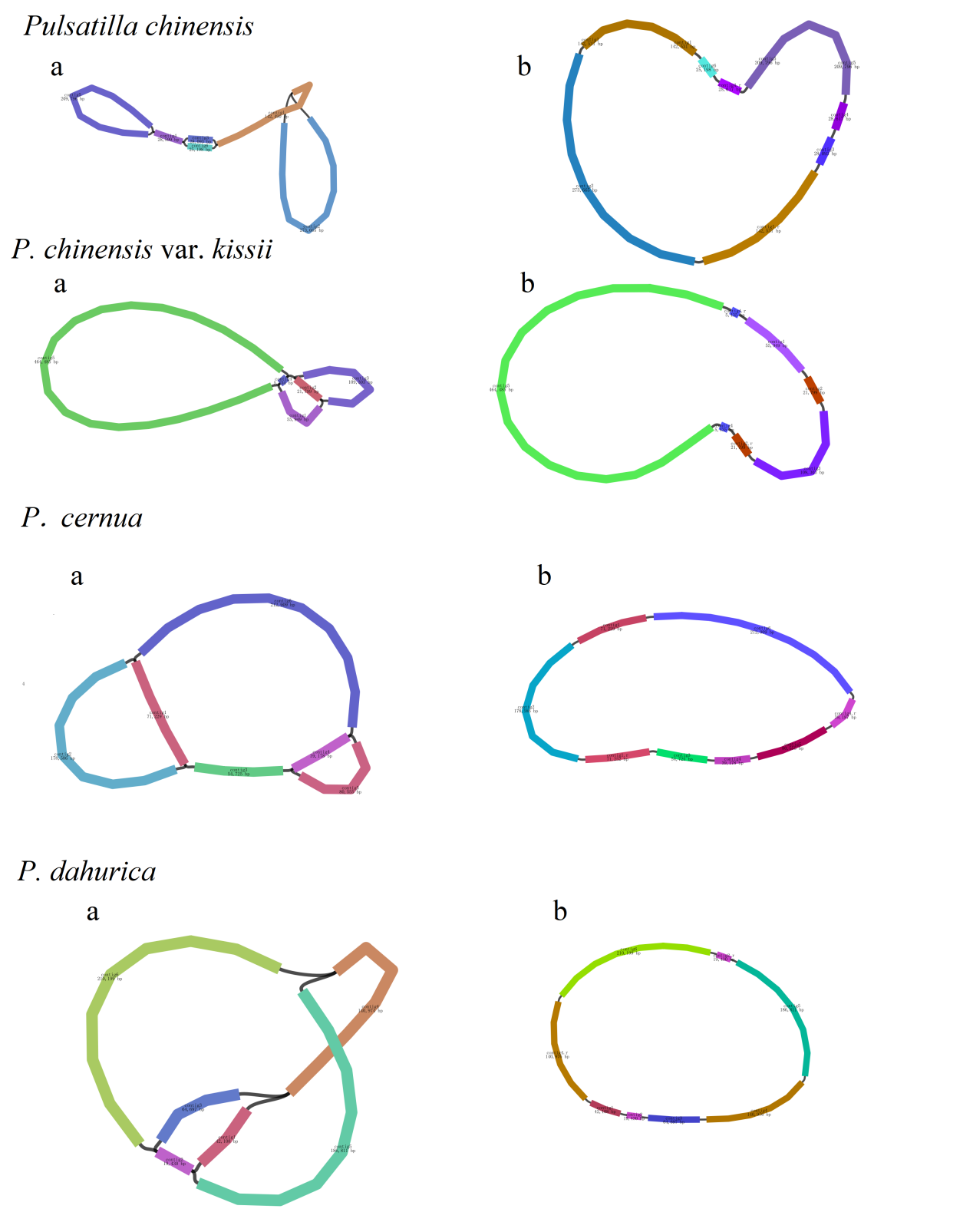


Fig. S3 The assembly graph of mitogenomes displayed in Bandage. (a) Raw assembly networks showing complex reticulation; (b) Curated circular structures inferred from repeat masking and validation


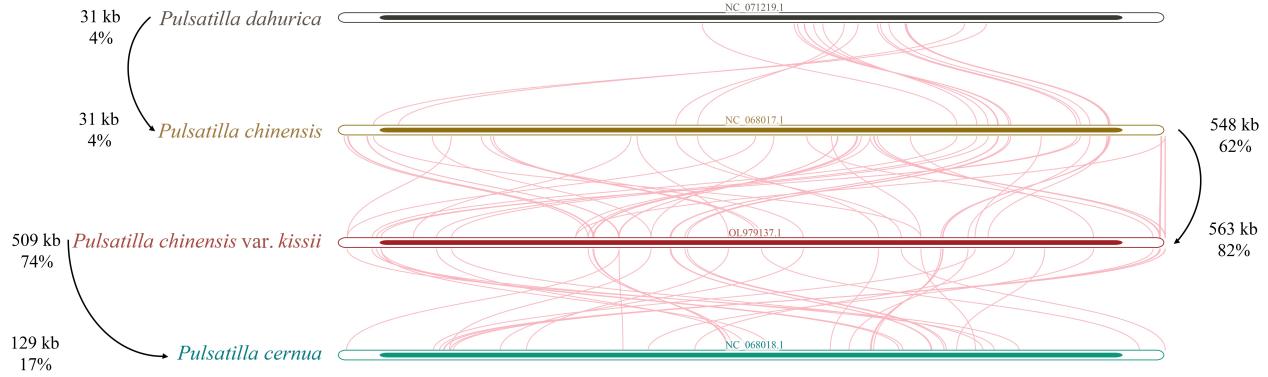


Fig. S4 Multiple synteny genes of the four mitogenomes


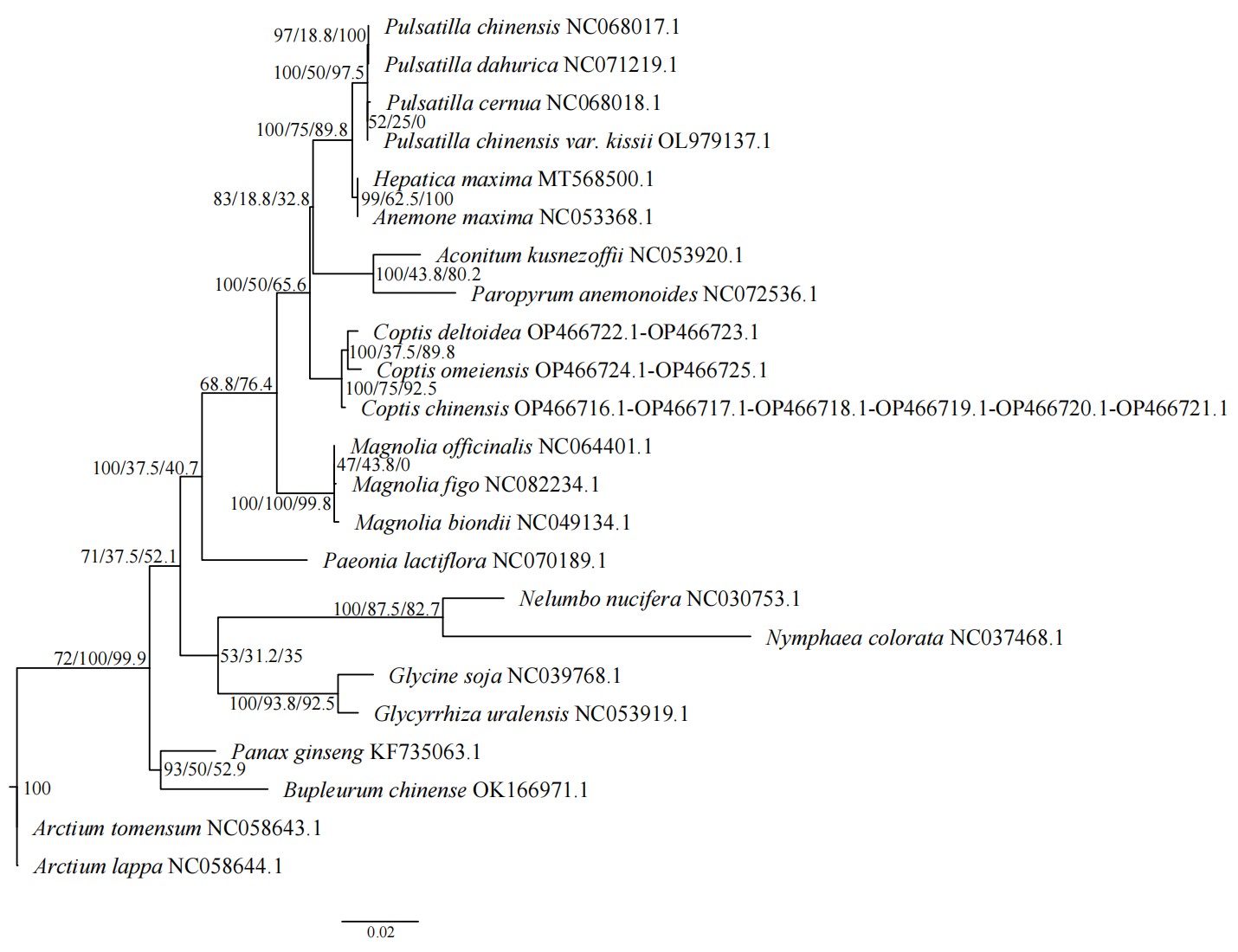


Fig. S5 Maximum likelihood Phylogenetic trees constructed using 14 PCGs of 23 mitogenomes. The tree were annotated with supports which were indicated by concordance factors (UFB/gCF/sCF)


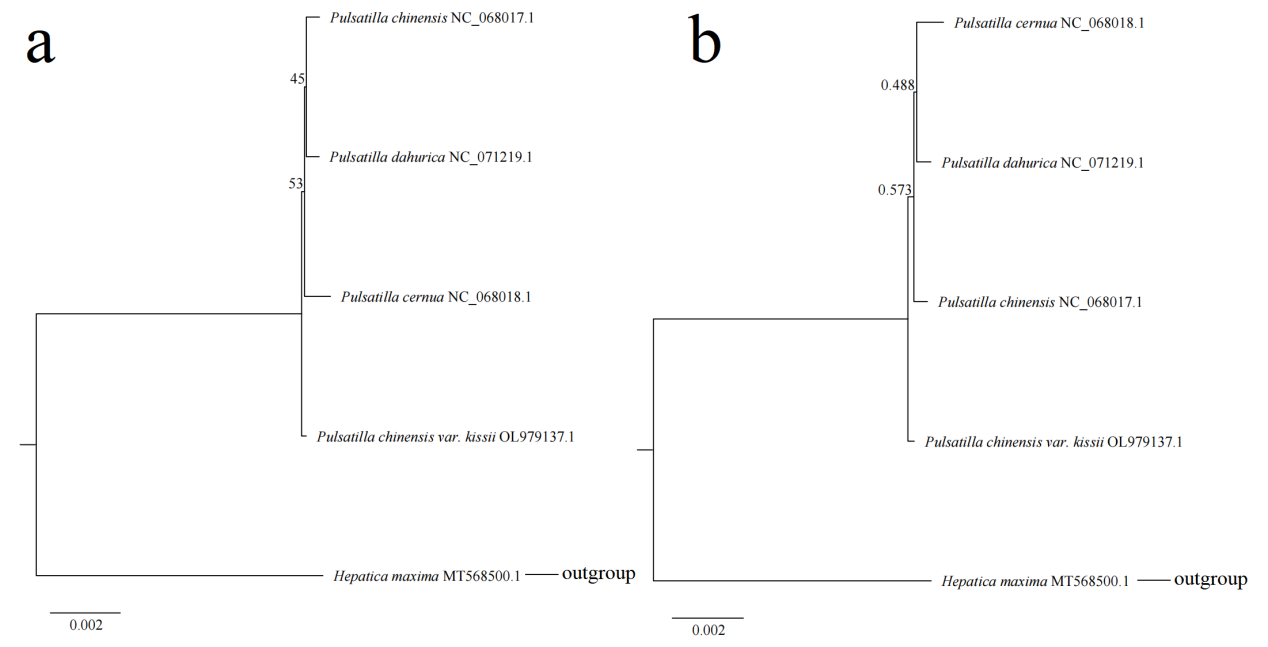


Fig. S6 Phylogenetic trees constructed using 21 PCGs (*atp4*, *atp6*, *atp8*, *atp9*, *ccmB*, *cob*, *cox1*, *cox3*, *nad1*, *nad2*, *nad4*, *nad4L*, *nad5*, *nad7*, *rpl2*, *rpl5*, *rpl10*, *rps4*, *rps11*, *sdh3*, *sdh4*) of 5 mitogenomes. (a) Maximum likelihood (ML) tree; (b) Bayesian inference (BI) tree
